# Nursing staff facilitate patient participation by championing the patient's perspective: An action research study in spinal cord injury rehabilitation

**DOI:** 10.1111/hex.13574

**Published:** 2022-08-25

**Authors:** Randi Steensgaard, Raymond Kolbaek, Sanne Angel

**Affiliations:** ^1^ Department of Neurology, Spinal Cord Injury Centre of Western Denmark Central Region Hospital Viborg Denmark; ^2^ Centre for Research in Clinical Nursing Central Region Hospital Viborg Denmark; ^3^ Department of Nursing Campus Viborg—VIA University College Viborg Denmark; ^4^ Research Unit for Nursing and Healthcare, Institute of Public Health Aarhus University Aarhus Denmark

**Keywords:** action research, care, nurse–patient relationship, nursing, patient participation, rehabilitation

## Abstract

**Introduction:**

Persons with spinal cord injury have experienced a life‐changing event, and they need to engage in the rehabilitation process to adjust to their current situation and future living conditions. Due to the highly contextual and varying psychological and physical ability to participate from patient to patient during rehabilitation, this is difficult for the injured person and for health professionals to support. Therefore, the aim of the study was to develop and facilitate patient participation by engaging nursing staff and from this engagement in the process, disclose methods to support participation.

**Methods:**

The processes conducted were based on an action research approach, from problem identification to the development, test and evaluation of four new nursing initiatives. The initiatives were developed by eight nursing staff members who participated actively as co‐researchers in a 2‐year study conducted at a Spinal Cord Injury Centre in Denmark from 2016 to 2018. Data evolved from workshops, transcriptions of meetings and written evaluations and was further analysed using Ricoeur's phenomenological‐hermeneutic approach.

**Results:**

Action research processes facilitated the development of four communicative initiatives and a shift in the nursing staff's support of the patient. In a collaborative process, the nursing staff acted as participants in the patient's rehabilitation. Awareness of the patient's perspective facilitated a caring, attentive and engaged approach from the nursing staff, which promoted rehabilitation tailored to the individual.

**Conclusion:**

Patient participation was enhanced when nursing staff actively participated in the development of initiatives and a culture supporting a person‐to‐person approach involving the patient and themselves as equal participants in the collaborative rehabilitation process.

**Patient or Public Contribution:**

Eight nursing staff members from the rehabilitation centre participated throughout the study as co‐researchers. Patients participated in observations and as informants in interviews during the first phase to identify challenges to patient participation. Patients also participated in testing the nursing initiatives during the action phase (Phase 3). Furthermore, a former patient was a member of the advisory board.

## 1 INTRODUCTION

Patient participation is recognized worldwide as a prerequisite for the quality of care, treatment and rehabilitation.^1^, ^2^, ^3^, ^4^ Benefits like patient safety, lower costs and higher quality of healthcare have political attention and have motivated health services to enhance patient participation.^5^, ^6^, ^7^, ^8^

In spinal cord injury (SCI) rehabilitation, patient participation is highlighted as a key to successful rehabilitation.^9^, ^10^, ^11^ This is due to the patient's struggle to cope with the far‐reaching consequences of an SCI on physical, psychological, social and existential levels.^12^, ^13^, ^14^, ^15^

The, often sudden, disruption of an individual's life has wide‐ranging consequences, and his or her work life, family life and social life may change forever, which may lead to a lower quality of life^16^ and/or severe psychiatric conditions.^17^, ^18^, ^19^ What previously counted as core elements of the lived life may need to be redefined to achieve a sense of continuity and meaning in life.^12^, ^20^

Therefore, the patient's sensemaking of the connections between past, present and future life is central to rehabilitation, where the focus is to ‘…enable persons with disabilities to attain and maintain their maximum independence, full physical, mental, social and vocational ability, and full inclusion and participation in all aspects of life’.^5^ The importance of the patient's participation is also central in the International Classification of Functioning, Disability and Health's (ICF) biopsychosocial approach to rehabilitation.^8^ This recognized approach includes the component ‘participation’ focusing on ‘the lived experience in the actual context in which people live’.^21^

Despite the growing body of literature on patient participation, research shows it is difficult to achieve individualised patient participation due to highly contextual and varying psychological and physical ability to participate from patient to patient during rehabilitation.^1^, ^22^, ^23^, ^24^, ^25^, ^26^

Attempts have been made to strengthen patient participation by using goal setting^27^ and shared decision making.^28^ Even so, the patient's everyday life and emotional issues lack attention.^27^

It has been suggested that the attitudes and approach of the interdisciplinary health professionals play a decisive role in the efforts of involving patients in healthcare and rehabilitation, which, in turn, affect implementation efforts.^10^, ^23^, ^29^, ^30^ This may be one of the reasons why health professionals struggle to implement the results from other studies into their own settings,^31^, ^32^, ^33^ urging the need for a different methodological approach to support the patient's participation.

Several studies argue that involving health professionals is a promising way of dealing with the barrier of implementation and changing health care practice.^28^, ^32^, ^34^ A possible methodological approach to overcome this challenge can be found in action research. It differs from traditional research by combining the act of changing practice with research while involving participants actively in the study.^34^, ^35^, ^36^, ^37^

Therefore, the aim of the study was to develop and facilitate patient participation by engaging nursing staff and from this engagement in the process, disclose methods to support participation.

## 2 MATERIALS AND METHODS

Based on its capacity to identify the issue and establish changes in practice,^38^, ^39^, ^40^ an action research design was applied to structure the approach of this study. The methodology of action research was inspired by Dewey's pragmatic philosophy.^39^, ^41^, ^42^ Hence, new insights, knowledge and skills were developed through a dynamic movement between experience, reflection and critical awareness of habits.

In four phases, nursing staff explored their existing and common traits. Accordingly, new awareness led to change through (1) identification of the local problem with patient participation, (2) development of four communicative nursing initiatives to support patient participation, (3) test and finally, (4) evaluation of the initiatives.

Iterative processes^43^, ^44^, ^45^ were supported by reflective writing in log‐books, reflective dialogues in workshops and meetings and action in practice (illustrated in Table 1).

**Table 1:** Four phases, aims, participants and methods

Phase	Aim	Participants	Methods
1: Constructing	To identify the shared meanings and challenges related to the issues of patient participation	Patients	A. Case studies (interviews with patients, co‐researcher log‐books, observations by the principal investigator) B. Workshop C. Consecutive meetings
		Co‐researchers	
		Supervisors	
		Principal investigator	
2: Planning	To collaborate on planning actions to address the identified issues.	Co‐researchers	B. Workshop C. Consecutive meetings
		Supervisors	
		Principal investigator	
3: Acting	To intervene and act upon identified issues while learning from the consequences.	Patients	B. Workshop C. Consecutive meetings D. Testing of four initiatives
		Co‐researchers	
		Supervisors	
		Principal investigator	
4: Evaluating	To evaluate the actions and discuss how they solved the issues of patient participation.	Co‐researchers	B. Workshop C. Consecutive meetings E. Prototype evaluations
		Supervisors	
		Principal investigator	

### 2.1 Setting and participants

The study was conducted at a Spinal Cord Injury Centre in Denmark, which is one of two national rehabilitation centres in Denmark where patients who have sustained SCIs are admitted for periods of 3–9 months. They are offered care, treatment and rehabilitation by an interdisciplinary team of health professionals, including physicians, physiotherapists and occupational therapists, psychologists, social workers and nursing assistants with 3 years of education and registered nurses.

An open invitation was accepted by eight nursing staff (four registered nurses and four nursing assistants) from a group of 55 nursing staff. Their experience in rehabilitation varied from 3 months to 19 years. They were all women and they all participated on equal terms. Being an explorative study with the methodological approach of action research, the co‐researchers were informed of their active role. The topic of patient participation was provided in advance, but the content, actions and knowledge evolved during the collaborative processes. The nursing staff members functioned as co‐researchers in all four phases of the study. Their participation was organised so they could act as co‐researchers as part of their normal working hours. Patients were not enroled as co‐researchers because the nursing staff members should be able to speak freely. Furthermore, the patients would have been discharged during the study because the study period was longer than the patients' hospitalization. Nevertheless, 11 patients were observed and interviewed by the first author in the first phase to identify challenges to patient participation as perceived by them. The interviews were analysed by the researchers using Ricoeur's text analysis. The findings from the interviews were part of the workshops with the co‐researchers. Patients also participated in testing the initiatives during Phase 3, the action phase.

The study had an organizational anchoring with an advisory board representing the co‐researchers, a former patient, the supervisors and the interprofessional managers as well as a representation from the head of the department. The aim of the board was the coordination of the project in accordance with the day‐to‐day administration of the centre. Furthermore, the board supported the co‐researchers' work and the implementation of the findings of the project. While not being directly involved, the interdisciplinary team members were continually informed throughout the entire project.

### 2.2 Data collection

Data were collected from 2016 to 2018 and consisted of co‐researchers' log‐book notes and the first author's observations from 19 days and 11 interviews with patients, four 1‐day workshops and nineteen 1‐h meetings held on a regular basis throughout the processes (Table 1). All interviews, meetings and workshops were audio‐recorded and transcribed verbatim. Finally, data were included from the co‐researchers' evaluations of the four communicative nursing initiatives based qualitative on open‐ended questionnaires.

### 2.3 Data analysis

Artistic, creative activities within the workshops sparked externalization of and reflection on tacit basic assumptions and habits. This facilitated access to experiences on both personal and group levels^46^, ^47^, ^48^, ^49^, ^50^, ^51^ and led to the deployment of four communicative nursing initiatives.

To achieve a surplus of meaning and to obtain a deeper understanding of what was immediately perceived in the action research processes, all transcriptions were analysed within the hermeneutic‐phenomenological tradition guided by Ricoeur's approach to text analysis.^52^, ^53^ The empirical material consisted of different sources as described in the section on data collection. To systematically and transparently bring out central findings from this diverse material, the same analytical approach was used regardless of the source. According to Ricoeur,^52^ all social phenomena of a semiotic nature can be analysed by using the text model. Therefore, the recorded interviews and dialogues from the workshops and meetings were transcribed into text and gathered with the rest of the data into one document. The analysis had three interrelated steps and the process moved back and forth to refine and strengthen the analysis. During the first step, the naïve reading, we read to obtain an immediate sense and impression of the text, leading to an overall interpretation of what the text said about patient participation. The next step, the structural analytical reading, was conducted as a line‐by‐line reading, where we moved our focus from what the text said to the meaning of the text in a broader sense and identified central themes. Finally, during the third step, critical analysis, we conducted an interpretation of what was the most probable understanding of what the text said about how the nursing staff supported patient participation.^52^ The analysis was performed by the first author under the support and supervision of the coauthors. It was also discussed with the co‐researchers. The quotations illustrate the findings of the analysis. The names are pseudonyms to keep anonymity as suggested by Eldh et al.^54^

### 2.4 Ethical considerations

The study was approved by the Danish Data Protection Agency (Journal no. 1‐16‐02‐503‐15).

The Danish Ethical Committee does not require approval for qualitative studies. Nevertheless, the Helsinki II Declaration and Ethical Guidelines for Nursing Research in the Nordic Countries were observed.

The study was approved by the rehabilitation centre and the Department of Neurology.

Participation in the study was voluntary for all participants and the patients and co‐researchers gave oral and written consent after receiving information and having the opportunity to ask questions about the study and its implications. An agreement was made with a psychologist whom the participants were able to consult if they felt psychological discomfort or harm during their participation.

## 3 FINDINGS

Through the action research processes, the nursing staff realised how knowledge about the patients' perspectives increased their ability to support patient participation. They developed and tested four nursing initiatives and found them effective as a method to facilitate patient participation.

**Figure 1:**
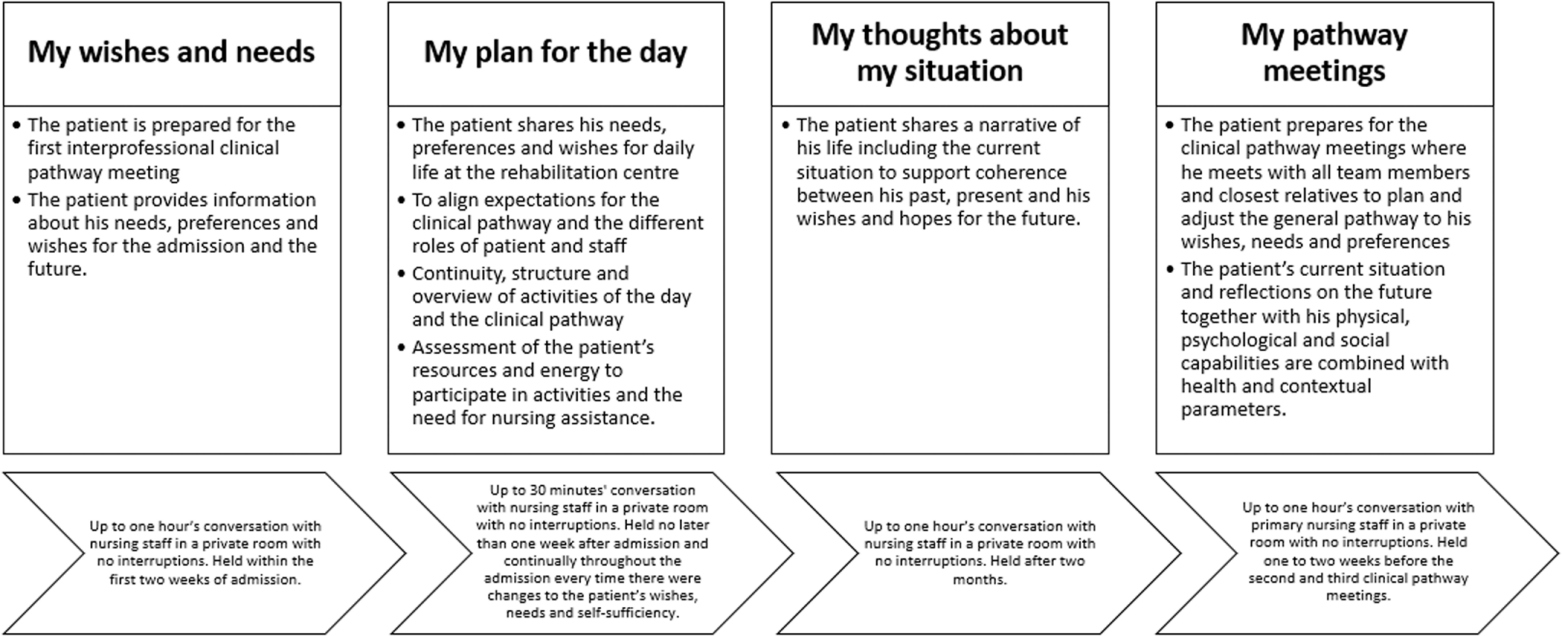
The aim and setting of the four communicative nursing initiatives developed by the co‐researchers.

A common feature of all the four communicative nursing initiatives (Figure 1) was that they provided structure and support to conversations between the patient and the nursing staff. They highlighted the perspective of the patient and promoted the patient's agenda over that of the nursing staff. Therefore, all the guides start with ‘My…’. The initiatives work independently but are linked and comprise the full rehabilitation process with variations in content and focus. All initiatives are described in guidelines, which include instructions for the nursing staff.

From the analysis of all materials, there are signs of a changed approach and attitude to patient participation in SCI rehabilitation. The nursing staff included the patient's perspective and experienced how this improved the planning of and patient engagement in the rehabilitation process. By being caring and attentive with an engaged approach, the nursing staff found themselves as participants in the patient's rehabilitation. The insights on how to support the patient's participation are elaborated on below in three interrelated themes:

### 3.1 Awareness of the patient's perspective facilitates a caring, attentive and engaged approach

When the nursing staff learned about their patients' perspectives, they felt compelled to form a closer relationship as a basis for providing care. Getting to know their patients as people, the nursing staff became sensitive to their patients' situations and felt engaged on a more personal level. This consolidated a person‐centred care approach as the basis for rehabilitation nursing. Recognizing the core value and original vocation as a caregiver, a co‐researcher expressed:

*We need to get back to our core job as nurses and certified healthcare workers. That is forming relationships and caring for our patients; once we have got that right, we can assist with bowel management (…)*.

Lise, co‐researcher, Meeting 17

A person‐centred care provision became the new overarching foundation for rehabilitation nursing. Listening attentively to the patient's perspective created space for thoughts, concerns and a new form of collaboration. The nursing staff experienced how this improved the patients' wellbeing:

*She said that it felt good getting the chance to tell her story; it was such a relief that somebody had the time to listen to her. This was also why she embarked on a psychological journey, because she had to think about and verbalise how she was feeling (…) this was the first time that she felt anyone had time for her and showed that they cared*.

Ann, co‐researcher, Meeting 6

The patient's description of relief was linked to the opportunity to share her experiences. This helped her make sense of her situation. Furthermore, the nursing staff's genuine interest in their patients' lives and wellbeing was experienced as caring. This made the nursing staff feel valued and raised their spirits.

### 3.2 Attentiveness to the patient's perspective and pre‐SCI life guide the rehabilitation pathway

In addition to facilitating care, the nursing staff's engagement seemed to support the patients' reflection on their situation. The patients had the opportunity to explain who they were, how they were feeling and what they felt about their current and future situations. This fixed point in the current process was important for the patients, as it represented the point from which their rehabilitation became meaningful to them. The nursing staff, as listeners and companions, helped to identify valuable elements from the patients' pre‐SCI lives. They helped their patients to understand what had happened and how to connect to their pre‐SCI lives and their lives to come. A co‐researcher describes how the conversations broadened the patient's perspective and how the patient gained a deeper self‐understanding, which aided the rehabilitation process:

*During conversations with patients, I often experienced how at first the patient focused on what had happened or what was difficult, but I also observed how patients ‘got through to the other side’ and were able to verbalise what was worth looking forward to, their progress and not least the hopes they had*.

Rikke, co‐researcher, evaluation of the four initiatives

When the nursing staff became attentive to the patient's perspective, they realized its absence from the planning of the SCI rehabilitation pathway as well as in the activities conducted during the process. Therefore, the nursing staff grasped the necessity of making time and space for the patients to share their previous routines and habits. In addition, the nursing staff experienced the importance of encouraging patients to share personal perspectives related to their situation and possible consequences for their lives in relation to family, work and social activities. When the patients shared their perspectives on their current situation in relation to their wishes for the future, their requirements for care and rehabilitation became clearer. As a co‐researcher puts it:

*We have had a deeper understanding since our conversation, as the background and his personality started to become clear. I can use that going forward when we are organising activities and more specifically how I guide, encourage and motivate the individual patient to get as far as possible in the rehabilitation process and to translate that into the bigger perspective, learning to live with a spinal cord injury*.

Hanne, co‐researcher, evaluation of nursing initiative

This quote illustrates how the nursing staff acknowledged the pivotal position of the patient's prior life, views, values and wishes. Thus, it became clear that getting access to patients' narratives and views of their situation was crucial to plan, adjust, clarify and negotiate a more individual and personalized rehabilitation process. Accordingly, the patient's perspective became a necessary component in planning rehabilitation.

### 3.3 Developing a collaborative process: The nursing staff as participants in the patient's rehabilitation

When the nursing staff were noticing the importance of providing more personal care, the issue of ‘who participates in what’ arose. Accordingly, the nursing staff expanded their view on patient participation in rehabilitation, from solely focusing on how the patient should participate to also include his/her own participation in the patient's process. One of the co‐researchers described this as taking part in the patient's journey:

*‘Our collaboration is characterised by the knowledge that we have together but also the difficult journey that we have been through*.

Annett, co‐researcher, evaluation of nursing initiative

This co‐researcher felt as if she was walking alongside the patient, not leading but accompanying the patient in a supportive and caring manner. The feeling of a shared task promoted engagement and a relationship built around enhancing the patient's ability to move forward.

Getting to know the patient's perspective, the nursing staff became aware that they needed to participate differently in the patient's rehabilitation process. Their participation was welcomed by the patients who in return opened up even more and shared their thoughts and feelings about their situation. This led to more profound engagement and the care provided by the nursing staff became much more attentive.

Another co‐researcher explained how the new approach brought out the patient's personality, giving him the opportunity to voice his needs and the motivation to move on. The co‐researcher described this as an opportunity to decipher the code for individualized rehabilitation or—to use a common metaphor—to help find the pieces of the puzzle for a coherent life:

*This is where the code for the patient's drive, his motivation, energy, problems, doubts and frustrations may come to the surface. Here the pieces of the puzzle are inspected to see what will fit, and maybe a few of the pieces are positioned, but that is not the aim. The aim must be to find the pieces and start the puzzle*.

Hanne, co‐researcher, evaluation of initiatives

The metaphor of a puzzle illustrates how the nursing staff perceived the new ‘picture’ that led to a new approach. A puzzle is complete when all the pieces are in place. However, in the case of rehabilitation, it is not up to the nursing staff to do the puzzle. It is not even to provide the pieces. Instead, it is to bring the patient's own pieces into play and do the puzzle together with the patient. The nursing staff's participation in completing the puzzle helped the patients to discover their own pieces.

## 4 DISCUSSION

The present study revealed that new communicative initiatives helped nursing staff to facilitate patient participation in rehabilitation. At the core of these initiatives was an openness to include the patient's perspective in the rehabilitation process. This implied that the nursing staff learned about the patient's life situation and understood how they could support the patients in their hopes and wishes regarding their rehabilitation and their future.

According to Wade^55^ rehabilitation is a person‐centred process tailored to the individual patient's needs just as personalised monitoring of changes is associated with interventions tailored to the needs, goals and wishes of the individual patient. The importance of a person‐centred approach to succeed in patient participation is widely recognized by health care professionals in general^9^, ^27^, ^55^, ^56^ and nurses in particular.^2^, ^3^, ^4^, ^57^ However, in a systematic review, Yun and Choi^58^ find that person‐centred care has not yet been implemented and fully adopted in rehabilitation settings. Furthermore, person‐centred care, as it has been reported so far, primarily focuses on goal‐setting and shared decision‐making. This can be problematic because patients with SCI can have a reduced ability to participate in shared decision‐making in the early phase of rehabilitation, which implies a need to balance autonomy and support.^10^ Combined with the need to secure respect and dignity^11^ the balance is difficult and challenges the person‐centred approach. Goal setting is still widely used^27^, ^55^ and considered to be good for making person‐centred rehabilitation.^59^ Our findings are not in opposition to goal setting. In fact, the consecutive communicative initiatives can spark the patients' reflections about their situation and ultimately, they can be transformed into individualised goals. Further, the nursing initiatives may become one of the nursing staff's methods to assist the patient in articulating their own wishes and needs when setting goals together with the interdisciplinary team.

The present study shows that being present and listening to the patient's wishes and needs provided the nursing staff with specific and individual knowledge about the patient as a person. Ultimately, the nursing staff got a clearer understanding of how the patients could participate in processes linked to his or her life. This confirms the importance of the patient's perspective, which was also found in one of few studies in SCI rehabilitation addressing the patients; perspective on patient participation and linked patient participation closely to person‐centred care.^11^ In this study, we present results of a changed understanding of the importance of the patient's perspective and we show signs of a changed approach where nursing staff participate in the patient's process. According to Martinsen,^60^ nursing staff must have an understanding and insight of the patient as a person, rather than merely as a patient to ‘participate in the world of the other’. According to Martinsen,^61^ this can occur when nurses are ‘sensing’ and ‘being’ with the person rather than focusing on producing and solving tasks. This new position in the relationship focused on the nursing staff's attention to the patient's perspective and facilitated a positive cycle that enhanced caring and provided a mutual engagement. This change of approach shows similarities to the concept of person‐centred practice, which according to Yun and Choi^58^ is an essential component for the quality of care in rehabilitation. Core values of person‐centred practice are described in the theory of McCormack et al.^62^ as respect for personhood, sharing autonomy, being authentic, being therapeutically caring, promoting healthfulness, showing respect for and actively engaging with the person's preferences, abilities, goals and lifestyle.

The collaborative process, also emphasized by Negrini et al.,^56^ was facilitated by communicative initiatives in the present study. The initiatives were developed by the nursing staff and therefore adjust to SCI rehabilitation in a specific context. This showed promising results because the nurses were not told what would be effective. They experienced it.

Hence, the methodology of this study involved the nursing staff and they developed a caring, attentive engagement. This helped them back to the cores of nursing and sparked their engagement. This attention to creating space for nursing staff to develop their nursing and perform nursing and care may result in a healthful culture, which is described as an important factor in successful person‐centred healthcare.^62^ Further, it may help nursing staff to find their position and contribution to the interprofessional team in rehabilitation, which is documented to be difficult.^63^, ^64^, ^65^, ^66^, ^67^, ^68^ Accordingly, McCormack et al.^69^ show how this essential, yet overlooked and deprioritized aspect of person‐centred care may improve the implementation of the approach. Interestingly, our findings show that even within the existing, fixed time logic and overall organization of the centre, the nursing staff were able to change their approach, learn about the patient's perspective and increase patient participation. This highlights the way in which we organized our study: We developed an environment for dialogue and reflection and cared for the well‐being of the nursing staff. We provided space for their perspectives to evolve.

In that sense, we took it a bit further than just acknowledging the need to listen. The nursing staff took the role of participants in an aspect of the patient's life and personal wishes and needs.

The findings of our study show a detailed picture of the power of lived experience and how this proved to increase the personal engagement of the nursing staff in their patients' pathways in a caring way. Therefore, involving nursing staff in developing and testing new procedures may be powerful and ultimately change their approach, attitude and way of working in a more person‐centred direction.

### 4.1 Strengths and limitations

The social complexity of action research limits the opportunity to create solutions and results that can be transferred directly to other contexts.^39^, ^70^ Therefore, transparency is crucial for others to be able to evaluate how to apply the results of this type of research.^71^

With large materials and many processes, this is difficult. Supplementing the findings of the action research processes with the application of Ricoeur's^52^ text model provided an opportunity to achieve further insight in addition to local development of knowledge and solutions hoping to increase the travelling capacity concerning how the local knowledge can attain value and rigour to be recognizable and usable in other settings and communities.^72^

Working with the local staff's attitudes and approaches to facilitate patient participation led to their renewed understanding and knowledge. However, we only had the opportunity to involve 8 out of 50 nursing staff members at the centre in the processes, which limited the personal involvement to a section of the entire group. The participating nursing staff actively applied for participation and they chose to participate out of interest with the risk of not being representative of the larger group.

Even though rehabilitation is an interdisciplinary task,^55^ we chose only to include nursing staff in this study. Therefore, the findings only reflect the nursing contribution to rehabilitation.

In rehabilitation, the use of ICF as a reference system is central.^73^ The ICF was not an explicit frame, but by listening to the patient thoughts and needs, different biopsychosocial elements were automatically touched. Nevertheless, this could be more interesting to explore further.

Furthermore, the time frame of the present study prevents the presentation of long‐term possibilities and consequences of the nursing initiatives and the approach adopted by the participating nurses, and we recommend further research on these aspects.

## 5 CONCLUSION

When nursing staff spend time engaging in the patient perspective, they are able to participate in a collaborative process tailoring rehabilitation to the individual patient's perspective, using his narrative, values and needs as the new focal point for personalised care. Accordingly, their commitment to understanding the patient's situation unfolds on a person‐to‐person level, which helps the nursing staff to engage with the patient and support him in his efforts to build a coherent life post‐SCI. This study shows how the caregivers' approach changes as a consequence of their first‐hand experiences. Including nursing staff directly in developing a person‐centred practice with the purpose of enhancing patient participation may lead to a changed approach where the attentiveness and awareness of the patient's perspective becomes a natural part of rehabilitation planning.

## CONFLICT OF INTEREST

The authors declare no conflict of interest.

## Data Availability

The data that support the findings of this study are available on request from the corresponding author.
